# Predominance of multi-resistant gram-negative bacteria colonizing chronic lower limb ulcers (CLLUs) at Bugando Medical Center

**DOI:** 10.1186/1756-0500-7-211

**Published:** 2014-04-04

**Authors:** Nyambura Moremi, Martha F Mushi, Mbunda Fidelis, Phillipo Chalya, Mariam Mirambo, Stephen E Mshana

**Affiliations:** 1 Department of Microbiology/Immunology, Catholic University of Health and Allied Sciences, P.O.BOX 1464 Mwanza, Tanzania; 2 Department of Surgery, Catholic University of Health and Allied Sciences, P.O.BOX 1464 Mwanza, Tanzania

**Keywords:** Chronic lower limb ulcers, Multi-drug resistant, Gram negative enteric

## Abstract

**Background:**

Infections, trauma, malignances and poorly controlled diabetes are common causes of chronic lower limb ulcerations in developing countries. Infected wound with multi-drug resistant bacteria usually are associated with increased morbidity and mortality. We report the distribution of bacteria pathogens colonizing the chronic lower limb ulcers and their drug susceptibility pattern from Bugando Medical Centre (BMC) a tertiary hospital in Tanzania.

**Findings:**

Three hundred non-repetitive wound swabs were aseptically collected from 300 patients with chronic lower limb ulcers using sterile swabs and processed following standard operative procedures. Isolates were identified using in house biochemical testing and in case of ambiguous confirmation was done using API 20NE and API 20E. Susceptibility was determined using disc diffusion test following clinical laboratory standard Institute guidelines (CLSI). Of 300 swabs from patients with chronic lower limbs ulcers, 201 (67.7%) had positive aerobic culture within 48 hours of incubation. Of 201 isolates, 180(89.6%) were gram-negative bacteria. Out of 180 gram negative bacteria, resistance was detected for ampicillin (95%, n = 171), amoxicillin/clavulanate (83.9%, n = 151), trimethoprim-sulphamethoxazole (78.9%, n = 142), ceftriaxone (46.7%, n = 84), ceftazidime (45.6%, n = 82), gentamicin (39.4%, n = 71), ciprofloxacin (17.8%, n = 32) and meropenem 28(15.6%, n = 25). A total of 41 (35%) of enterobacteriaceae were found to be extended spectrum beta-lactamases (ESBL) producers while of 18 *Staphylococcus aureus,* 8(44.4%) were found to be methicillin resistant *Staphylococcus aureus* (MRSA).

**Conclusion:**

There is high prevalence of ESBL and MRSA isolates in surgical wards at BMC. We recommend infection control and antibiotic stewardship programs in these wards to minimize spread of multi-resistant organisms.

## Findings

### Background

In developing countries infections, trauma, malignances and poorly controlled diabetes are the most common causes of chronic lower limb ulcerations [1,2]. An infected wound complicates the postoperative course and results in prolonged hospital stay and delayed recovery [3]. Prolonged hospital stay usually exposes the patient to health care associated infections (HCAs) [4], and more risk to infection due multi-drug resistant bacteria like extended spectrum beta lactamase (ESBL) producers and methicillin resistant *Staphylococcus aureus* (MRSA) [5-8]. In Tanzania about 50% of *Klebsiella pneumonia* and 25 -45% of *Escherichia coli* isolated from HCAIs are reported to be ESBL producers [9-11]. Also in Tanzania *S. aureus* has been reported to be the commonest cause of surgical site infections of which 18.8% are reported to be MRSA [3].

Clinical experience and outcome of patients regarding chronic lower limb ulcers in this centre has been described in previous publication [12]. This article is building from the same study but focusing on distribution of bacteria pathogens, susceptibility pattern of gram negative and gram positive isolates from chronic lower limb ulcers. In addition more information regarding the susceptibility pattern that predicts ESBL phenotype is presented.

The predominance of multi-drug resistant gram negative bacteria colonizing and infecting lower limbs necessitates the scaling up of infection control practices and the introduction of antibiotic stewardship in surgical wards in developing countries.

### Methods

#### Study population

A cross sectional study involving all patients with chronic lower limbs ulcers was conducted between November 2011 and February 2012 in surgical wards of Bugando Medical Centre (BMC), a 1000 bed capacity tertiary hospital in the northwestern part of Tanzania as previously described [12].

#### Laboratory procedures

A total of 300 non-repetitive wound swabs were collected from 300 patients with chronic lower limb ulcers using sterile swabs. All swabs were processed as previously described [13]. Briefly specimens were inoculated on MacConkey agar and 7% sheep blood agar (HIMEDIA, INDIA) and incubated at 35°C aerobically for 24-48 hrs. Identification of bacteria was done using colonies characteristics, hemolysis on blood agar, lactose fermentation on differential media and in house biochemical tests using colonies from pure cultures; in case of ambiguous results confirmation was done using API 20NE and API 20E [11].

#### Antibacterial susceptibility testing

Susceptibility testing was performed by Kirby-Bauer technique. The 0.5 McFarland of test organism was uniformly seeded over the Mueller-Hinton agar surface and incubated at 35°C for 24 hours. Interpretation was made as per CLSI [14]. Antibiotics discs tested for gram positive bacteria included ampicillin (10 μg), cefoxitin (30 μg), trimethoprim-sulphamethoxazole (1.25/23.75 μg), gentamicin (10 μg), erythromycin (15 μg), clindamycin (2 μg) and vancomycin (30 μg). For gram negative bacteria, disc tested include ceftazidime (30 μg), amoxicillin/clavulanate (20/10 μg), ceftriaxone (30 μg), gentamicin (10 μg), trimethoprim-sulphamethoxazole (1.25/23.75 μg), ciprofloxacin (5 μg), meropenem (10 μg) and ampicillin (10 μg). ESBL producers were detected using disk approximation method as described previously [15,16] while MRSA were detected using cefoxitin disc 30 μg, all isolates resistant to 30 μg cefoxitin based on CLSI were considered as MRSA [17]. In this study all isolates resistant to at least three of the following antimicrobials: ampicillin, amoxicillin/clavulanate, ceftazidime, ciprofloxacin, gentamicin, and/or trimethoprim-sulphamethoxazole (SXT) were considered as multi-drug resistant bacteria [18].

The study was approved by Bugando Medical Centre/Catholic University of Health and Allied Sciences ethics committee and the informed consent were obtained from all patients.

### Results

A total of 300 patients with CLLUs were included in the study. Traumatic ulcers were the most frequent type of ulcer accounting for 181 (60.3%) of patients followed by infective ulcers 43 (14.3%), metabolic ulcers 35 (11.7%), neoplastic/malignant ulcers 20 (6.7%), vascular ulcers 11 (3.7%), neuropathic ulcers 8 (2.7%) and ulcerating skin lesions e.g. pyogenic granulomatous 2 (0.7%). Out of 300, 201 (67%) had positive aerobic culture within 48 hours of incubation. Of 201 positive cultures 180(89.6%) were identified as gram negative bacteria and 21(15.9%) as gram positive bacteria (*Staphylococcus aureus* 18, *Enterococcus* spp 3). Bacteria species isolated were; *Pseudomonas* spp. 54 (26.9%), *Proteus* spp. 45 (22.4%), *Klebsiella pneumoniae* 33 (16.4%), *E. coli* 26 (12.9%), *Staphylococcus aureus* 18 (9%), *Acinetobacter* spp. 9 (4.5%), *Serratia* spp. 7 (3.5%), *Enterobacter* spp. 6 (3%) and *Enterococcus* spp 3 (1.5%).

Of 180 gram negative bacteria, resistance was detected for ampicillin (95%, n = 171), amoxicillin/clavulanate (83.9%, n = 151), trimethoprim-sulphamethoxazole (78.9%, n = 142), ceftriaxone (46.7%, n = 84), ceftazidime (45.6%, n = 82), gentamicin (39.4%, n = 71), ciprofloxacin (17.8%, n = 32) and meropenem 28 (15.6%, n = 25) Table 1. A total of 41 (35%) of enterobacteriaceae were found to be ESBL producers, with specific ESBL rate for *Escherichia coli* and *Klebsiella* spp being 50% and 53% respectively. The resistance to trimethoprim-sulphamethoxazole, gentamicin and ciprofloxacin was found to predict ESBL phenotype (Table 2). *Staphylococcus aureus* were resistant to penicillin (79.3%), amoxicillin/clavulanate (60%), gentamicin (8.3%), trimethoprim-sulphamethoxazole (60%), clindamycin (20.7%), erythromycin (13.8%) and 8 (44.4%) were found to be MRSA.

**Table 1:** Resistance pattern of 180 gram negative isolates to various antibiotics in percentage

**Bacteria isolates**	**N**	**AMP**	**CAZ**	**AMC**	**CRO**	**GM**	**SXT**	**CIP**	**MEM**
*E.coli*	26	100	50.0	88.5	50.0	30.8	76.9	19.2	19.2
*Klebsiella* spp.	33	100	59.4	93.9	59.3	54.5	78.8	9.1	6.06
*Pseudomonas* spp.	54	100	55.1	92.6	55.1	44.4	96.3	20.4	25.9
*Proteus* spp.	45	88.9	20.0	71.1	20.0	22.2	57.8	17.8	2.2
*Other gm–ve spp	22	80.0	45.9	68.0	54.5	52.0	84.0	24.0	28.0
Total	180	95	45.6	83.9	46.7	39.4	78.9	17.8	15.6

Keys: AMP = Ampicillin, CAZ = Ceftazidime, AMC = Augmentin, CRO = Ceftriaxone GM = Gentamicin, SXT = trimethoprim-sulphamethoxazole, CIP = Ciprofloxacin, MEM = Meropenem. * Acinetobacter spp., *Serratia* spp. and *Enterobacter* spp.

**Table 2:** Antimicrobial resistance pattern that predict ESBL phenotype

**Predictive drug resistance phenotype**	**ESBL n (%)**		**OR (95% CI)**	**p- value**
	**Yes**	**No**		
**AAGCS**				
Yes	12 (44.4)	15 (55.6)	3.50 (1.5-8.3)	0.004
No	29 (18.6)	127 (81.4)	1	
**AAG**				
Yes	30 (44.8)	37 (55.2)	7.74 (3.5-17.0)	<0.001
No	11(9.48)	105 (90.5)	1	
**AAC**				
Yes	14 (43.8)	18 (56.3)	3.57 (1.6-8.1)	0.002
No	27 (17.9)	124 (82.1)	1	
**GCS**				
Yes	12 (42.9)	16 (57.1)	3.26 (1.4-7.6)	0.006
No	29 (18.7)	126 (81.3)	1	
**GC**				
Yes	12 (42.9)	16 (57.1)	3.26 (1.4-7.6)	0.006
No	29 (18.7)	126 (81.3)	1	
**GS**				
Yes	33 (45.8)	39 (54.2)	10.89 (4.6-25.6)	<0.001
No	8 (7.2)	103 (92.8)	1	

Note: AAGCS = A- Ampicillin, A- Amoxicillin/Clavulanate, G- Gentamicin, C-Ciprofloxacin, and S- trimethoprim-sulphamethoxazole, AAG = A- Ampicillin, A- Augmentin, G- Gentamicin, AAC = A- Ampicillin, A- Augmentin, C- Ciprofloxacin, GCS = G- Gentamicin, C- Ciprofloxacin, S- trimethoprim-sulphamethoxazole, GC = G- Gentamicin, C- Ciprofloxacin, GS = G- Gentamicin, S- trimethoprim-sulphamethoxazole.

### Discussion

The microbiological profile of chronic ulcers of the lower limbs is very important in the provision of appropriate management of ulcers as well as institution-specific antibiotic policy in the surgical wards [19]. As reported previously [3], *Pseudomonas aeruginosa* was the most frequent gram negative bacteria isolated while *Staphylococcus aureus* was the commonest gram positive bacteria*.* Most of these isolates were multiply–resistant to commonly used antibiotics. This is due to the fact that most of these patients were hospitalized for more than 72 hrs signifying health care associated infections [3].

Compared to previous data in the surgical wards [3] the ESBL rates among *Klebsiella spp* and *E. coli* colonizing CLLU were lower than those involved in surgical site infections. Also this study confirmed the previous observation [10,11] that the resistance to trimethoprim-sulphamethoxazole, gentamicin, and ciprofloxacin is a predictor of ESBL phenotype. Most of ESBL producers in this study are multi-drug resistance, due to the fact that most of ESBL conjugative plasmids observed previously in this settings [20,21] carry resistance markers for other antibiotic classes such as tetracycline, gentamicin and trimethoprim-sulphamethoxazole. Similar to other studies [11,22], majority of gram negative and gram positive isolates were sensitive to meropenem and vancomycin respectively. Increased trend was observed for MRSA at BMC whereby in 2009 and 2011 about 16.3% and 18.8% of *S. aureus* were found to be MRSA respectively [3,17] while in the current study about 44% of *Staphylococcus aureus* were found to be MRSA.

Despite the importance of these data some limitations were failure to perform; anaerobic culture, molecular characterization and PCR confirmation of ESBL and MRSA phenotype.

### Conclusion

High prevalence of ESBL and MRSA isolates were observed in surgical wards at BMC. We highly recommend Infection control and antibiotic stewardship programs in these wards to minimize spread of multi-resistant organisms. Coordinated surveillance of multi drug resistant isolates in Tanzania and other developing countries is highly needed so that this worldwide public health problem is controlled.

## Competing interests

The authors declare that they have no competing interests.

## Authors’ contributions

MFM, NM, SEM participated in the design of the work, data analysis and interpretation of the results; FM, PC collected specimens; MFM, MM and SEM prepared the first draft of the manuscript; all authors read and approved the final manuscript.
